# SKGEL® Implant Versus T-Flux® Implant in the Contralateral Eye in Deep Sclerectomy with Phacoemulsification: Long-Term Follow-Up

**DOI:** 10.2174/1874364100802010057

**Published:** 2008-03-28

**Authors:** Frank Schreyger, Gabor Scharioth, Holger Baatz

**Affiliations:** Augenzentrum Recklinghausen, Erlbruch 34-36, D-45657 Recklinghausen, Germany

## Abstract

**Purpose:**

To intraindividually compare the efficacy and safety of the SKGEL^®^ implant versus the T-Flux^®^ implant in deep sclerectomy.

**Methods:**

In a retrospective analysis 17 patients were identified who underwent combined phacoemulsification-deep sclerectomy and implantation of SKGEL^®^ in one eye and T-Flux^®^ in the contralateral eye.

**Results:**

In eyes with SKGEL^®^ the IOP decreased from 20.6+7.3 mm Hg to 14.8+5.3 mm Hg (-5.8 mm Hg or -28.1%), and in eyes with T-Flux^®^ from 19.9+7.2 mm Hg to 14.7+3.3 mm Hg (-5.2 mm Hg or -26.1%, no statistically significant difference, p >0.05). Antiglaucoma medications with either implant decreased from initially 2.0+0.8 to 0.3+0.7. A qualified success was found in 17/17 eyes with T-Flux^®^ and in 16/17 eyes with SKGEL^®^. Complete success was achieved in 14/17 eyes with T-Flux^®^ and in 13/17 eyes with SKGEL^®^.

**Conclusions:**

The IOP-lowering effect and safety of SKGEL^®^ and T-Flux^®^ seem to be comparable.

## INTRODUCTION

Non-penetrating glaucoma surgery techniques such as deep sclerectomy or viscocanalostomy have been developed in recent years. These techniques are presented in a number of publications and are now considered to be as effective as trabeculectomy in lowering intraocular pressure (IOP), with the advantage of being associated with fewer complications [1-8]. Especially early complications, e.g. hypotony, hyphema and shallow anterior chamber are observed to a much lesser extent.

A space maintaining implant in the scleral bed seems to enhance the IOP-lowering effect of deep sclerectomy [1,9-11]. Several devices exist which are either absorbable, e.g. collagen and reticulated/nonreticulated hyaluronic acid or nonabsorbable, e.g. polymethylmethacrylate or Poly-Megma hydrogel, and are used with success in deep sclerectomy [12-18]. The combination of glaucoma and cataract surgery is advantageous in patients suffering from both glaucoma and senile cataract, avoiding a second operation in the same eye [19,20]. Combined surgery is considered safe and effective all the more as phacoemulsification alone is presumed to lower IOP [21,22], or might improve the effect of deep sclerectomy [23].

The aim of this retrospective analysis is to compare the IOP-lowering effect of the absorbable SKGEL^®^ with the nonabsorbable T-Flux^®^ implant. The intraindividual comparison limits the influence of individual variations in the inflammatory and fibrotic response. All eyes included in the analysis received simultaneous phacoemulsification, minimizing the impact of an additional IOP-lowering effect by phacoemulsification.

## METHODS

For this retrospective study a decided approval of an IRB/Ethics Committee was not required. The study was in compliance with the Declaration of Helsinki. From January 2000 to December 2006, 321 eyes of 189 patients were treated with deep sclerectomy and simultaneous phacoemulsification in the Augenzentrum (eye centre) Recklinghausen, Germany. Out of this number, a total of 17 patients with 34 eyes were identified who had received the absorbable device SKGEL^®^ 3.5 (Corneal Laboratories, Paris, France) in one eye and the nonabsorbable T-Flux^®^ device (IOLTECH Laboratories, La Rochelle, France) in the contralateral eye. The first of these 17 patients had his first eye operated in April 2002, the last patient his second eye in April 2006, so that postoperative IOP was followed for a maximum of 4 years and a minimum of 6 months. SKGEL^®^ 3.5 is a slowly absorbable implant consisting of a 35 µg crosslinked (reticulated) sodium hyaluronate. It is soft, transparent and has the shape of an equilateral triangle with a length of 3.5 mm per side and a thickness of 0.5 mm. T-Flux^®^ is a nonabsorbable implant made of highly hydrophilic acrylic, a Poly-Megma hydrogel. It is non-degradable, biocompatible and resistant to fibrosis. It is transparent, flexible and shaped like a T where the length of the arms is 4.0 mm, the body height is 3.4 mm and thickness is from 0.15 to 0.35 mm. It has a target hole for YAG-laser micropuncture and an additional hole for suture fixation of the implant. Assuming that both SKGEL^®^ and T-Flux^®^ should decrease IOP to the same extent and that both have the same safety profile, the surgeon had no preference for any of the devices to be implanted. Indication for surgery was medically uncontrolled primary open-angle glaucoma defined by an intraocular pressure (IOP) higher than 21 mm Hg under maximal therapy; or progression of visual field defects in standard automated perimetry or progression of the optic nerve excavation with or without elevated IOP, intolerance to antiglaucomatous drops, low patient compliance, as well as a clinically relevant senile cataract. Additional criteria to include a patient in the analysis were bilateral combined surgery with a postoperative follow-up of at least 6 months and no previously performed glaucoma surgery. In case of small, microscopic perforations of the trabeculo-Descemet’s membrane during surgery, the eye was nevertheless included, as long as the intervention  was completed as a deep sclerectomy. Written informed consent was obtained from all patients for both phacoemulsification and deep sclerectomy in one session. Prior to consenting all patients were informed that a device would be implanted, that several kinds of implants exist, and that the type chosen depended on the surgeon’s decision. Before surgery, all patients underwent the following examinations: best corrected visual acuity, full slit-lamp examination, fundus examination, gonioscopy, IOP with Goldmann applanation tonometer, standard automated static white-on-white 30° perimetry (Octopus 101, Peridata software, program G2, Haag-Streit, Switzerland). All combined operations were performed by one surgeon (G.S.). Normally both operations were carried out within 3 months, only in one patient the interval was longer than one year due to general health problems. Deep sclerectomy was made in the superior quadrant. A fornix-based conjunctival flap was created and the sclera exposed. A one-third scleral thickness limbus-based scleral flap of approximately 5 x 4 mm in size was marked using a 15° slit knife. The flap was dissected up to 1 mm into perilimbal clear cornea using a bevel-up crescent knife (1-mm ultrasharp knife, Grieshaber, Alcon). This was followed by nasal and temporal paracenteses and a clear corneal phacoemulsification in the same site through a 2.8 mm incision under the superficial scleral flap. A foldable acrylic intraocular lens was placed in the capsular bag. After IOL placement, glaucoma surgery was continued: A triangular smaller flap of deep sclera was dissected, leaving only a thin layer of scleral fibers over the choroid. The base of the flap was extended up to 1 mm into clear cornea and down to the Descemet’s membrane, unroofing Schlemm’s canal. The deep flap was excised with scissors, carefully avoiding tears in the Descemet. The inner wall of the Schlemm canal and the juxtacanalicular trabecular meshwork was peeled off with Utrata forceps. After establishing that aqueous humour percolates through the trabecular-Descemet’s membrane, either the SKGEL^®^ or the T-Flux^®^ implant was placed into the scleral bed without a suture. Both arms of the T-Flux^®^ device were carefully inserted into the openings of Schlemm’s canal. Both the superficial scleral flap and the conjunctiva were repositioned and tightly closed with 4-5 single absorbable sutures (10x0 Biosorb, Alcon). Postoperatively, all patients received topical corticosteroids (prednisolone acetate 1%) for at least 6 weeks. Postoperative visits were performed at one day, one week, one month, 3 months, 6 months, and depending on the date of surgery, for up to 4 years at least once a year. During these visits the following data were collected: IOP, best corrected visual acuity, existence of a filtration bleb, complications, and need for additional antiglaucoma therapy. Standard automated perimetry was carried out every 6 months. Follow-up was partly done by referring ophthalmologists outside the eye centre. At the end of the individual observation period, glaucoma surgery was considered a complete success when IOP was 21 mm Hg or less without antiglaucoma medication and a qualified success when IOP was 21 mm Hg or less with or without antiglaucoma treatment. The operation was considered a failure when IOP was higher than 21 mm Hg, or when further glaucoma surgery was needed or when deterioration of the visual function was detected. However, small interventions like needle revision or laser goniopunctures would not have been judged as failures.

Unpaired sample two-tailed Student’s t-test was used to compare the pre- and postoperative IOP measurements. A p-value of less than 0.05 was considered statistically significant.

## RESULTS

Of the 17 patients eligible for case analysis under the above mentioned criteria, 3 were male and 14 female with a mean age of 77.1 +6.8 years (± standard deviation). The overall mean follow-up period was 26.5 +16.4 months in the SKGEL^®^ group and 27.2 +16.0 months in the T-Flux^®^ group, with a range from 6 to 48 months in both groups. The mean preoperative IOP was 20.6 +7.3 in the SKGEL^®^ group and 19.9 +7.2 mm Hg in the T-Flux^®^ group and hence considered comparable (p >0.05).

In both groups the surgical intervention led to a clinically relevant result: In the SKGEL^®^ group the mean preoperative IOP decreased to a mean final IOP of 14.8 +5.3 mm Hg (-5.8 mm Hg or -28.1%), and in the T-Flux^®^ group to 14.7 +3.3 mm Hg (-5.2 mm Hg or -26.1%). The difference between both groups is statistically not significant (p >0.05). IOP results over the full observation period in both groups are displayed in Table **1**.

**Fig. (1):**
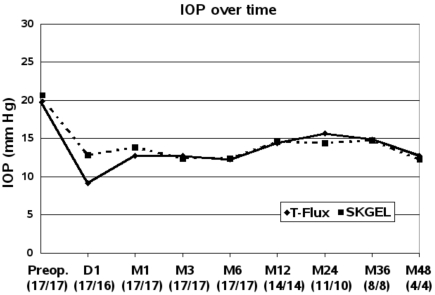
Intraocular pressure (IOP) course over time D = day, M = months, (number of eyes with T-Flux^®^ / SKGel^®^).

Four eyes in each group were followed up for up to four years, Three eyes in both groups for at least 6 months. After implantation of T-Flux^®^ or SKGEL^®^ the curves of IOP measurements in both groups were similar in the long-term (Fig. **1**).

The mean number of antiglaucoma medications before surgery was 2.0 +0.8 substances in both groups, with a minimum of one substance and a maximum of 4 substances. With both implants the need for antiglaucoma treatment decreased after surgery to 0.3 +0.7 medications at the final visit. In other terms, only 3 eyes per group required a permanent treatment after surgery, with a maximum of 2 substances. 2 eyes, one in each group, needed a transitory therapy with betablocker drops for a short time, and no further therapy was necessary in these eyes afterwards.

At the end of the observation period a qualified success (IOP < 21 mm Hg with or without treatment) was found in all 17 eyes with the T-Flux^®^ device (100%), and in 16 eyes with a SKGEL^®^ implant (94.1%). Complete success (IOP < 21 mm Hg without treatment) was achieved in 14 T-Flux^®^ eyes (82.4%) and in 13 SKGEL^®^ eyes (76.5%). One eye in the SKGEL^®^ group has to be considered a failure, as cyclokryocoagulation was inevitable 6 months postoperatively due to a strong increase in IOP which was refractory to any medical therapy.

Complications during glaucoma surgery were: microperforation of the trabeculo-Descemet’s membrane in 2 eyes in the T-Flux^®^ and in one eye in the SKGEL^®^ group. However, in all cases the deep sclerectomy with implantation of a device could be completed as planned. Apart from the usual non-serious intraocular inflammatory signs, in the early postoperative phase a massive hyphema was observed in one eye with a T-Flux^®^ implant, possibly due to a hypertensive crisis the patient suffered. The bleeding stopped spontaneously and was managed by anterior chamber irrigation. In the other group one eye with SKGEL^®^ had longstanding corneal erosion with corneal edema which might have been caused by the cataract operation. However, cataract surgery was done in all 34 eyes without any intraoperative complication. In all patients a foldable acrylic posterior chamber IOL was implanted. In the observation period no other severe complications related to the IOL occurred. There was no indication that visual acuity was worse in the eyes of these patients than in those who had a simple phacoemulsification without a combined deep sclerectomy in our centre. In some eyes an opacified posterior capsule was treated uneventfully by Nd: YAG laser capsulotomy. Visual field results did not reveal clinically relevant deteriorations apart from some fluctuation of scotomas, i.e. no increase in scotomas were detected in follow-up visual field tests. In one eye which was implanted with an SKGEL^®^ device and that had well-controlled IOP without antiglaucomatous therapy, a sharp increase in IOP was noted after approximately 6 months. The rise in IOP was due to neovascular glaucoma secondary to a central vein occlusion. Despite full medical treatment, the IOP remained uncontrolled with values over 30 mm Hg, and a decision was made to perform a cyclokryocoagulation, after which the IOP remained at low values throughout the following 4 years of observation without any treatment. This was the only case where surgical intervention was necessary after glaucoma surgery. No laser goniopuncture, no needling or other procedures, e.g. antimetabolite injections, were required. Most eyes had no filtering bleb, and only a few had a flat or diffuse filtering bleb.

## DISCUSSION

The mechanisms by which deep sclerectomy lowers IOP are still not fully understood [24]. However, deep sclerectomy has an effect on filtration, hence its success is vulnerable to scarring and will be positively influenced by the use of antimetabolites and theoretically by a device implanted within the scleral bed [24-26]. These so-called space maintainers are either absorbable implants which degrade within several months or nonabsorbable persisting devices [2,10,27,28]. Judging from our years of experience with implantation of the devices in glaucoma surgery, we assumed that both the absorbable and the nonabsorbable implant lower IOP approximately to the same level. Therefore we had no preference for a certain device. Also, there were no special indications or circumstances where we expected one device to perform better than the other. Therefore, over the years we accumulated some patients who required glaucoma surgery on both eyes and who happened to be implanted with both devices. Our retrospective analysis of the patients who received SKGEL^®^ in one eye and T-Flux^®^ in the contralateral eye bore the opportunity to compare both devices regarding their IOP-lowering effect. The intraindividual comparison limits the effect of confounding variables, e.g. the influence of individual variations in the inflammatory reaction or fibrotic response after deep sclerectomy. Moreover, all eyes underwent simultaneous phacoemulsification, precluding a systematical bias because of a possible IOP-lowering effect of phacoemulsification. Only very few studies with an intraindividual control have been performed up to now and only few studies have a comparably long follow-up with implants in deep sclerectomy. Furthermore, to our knowledge there are only two studies comparing the efficacy of absorbable versus nonabsorbable implants: Wiermann *et al*. [29] investigated the efficacy of SKGEL^®^ and T-Flux^®^ implants with and without phacoemulsification in a retrospective study of 241 patients over a period of 12 months. The results showed no difference in IOP in all 4 groups. Mansouri *et al*. [16] compared a nonabsorbable polymethylmethacrylate implant versus an absorbable collagen device for a mean observation period of 20 months. The effect on IOP and the rate of complications were the same in both groups.

Other studies compared T-Flux^®^ versus absorbable non-reticulated hyaluronic acid. Ravinet *et al*. [17] studied the effect of T-Flux^®^ versus Healon GV^®^. During a 2-years follow-up both treatments had fully comparable results. Ates *et al*. [12] compared deep sclerectomy with a T-Flux^®^ implant versus viscocanalostomy: The success rates were similar and not significantly better than those of viscocanalostomy. Detry-Morel [2] in her 5-year long-term study compared deep sclerectomy in combination with T-Flux^®^ / SKGEL^®^ with and without antimetabolite (5-fluorouracil or mitomycine) versus Healon GV^®^ and versus antimetabolite application alone. Mean IOP decrease revealed no statistically significant difference between the 4 groups.

Theoretically, in the long term absorbable implants like SKGEL^®^ might work less well than nonabsorbable devices. The implantation of a nonabsorbable device could create an intrascleral space by permanently preventing adhesion between the scleral flap and the scleral bed. Dahan *et al*. [30] carried out ultrasound biomicroscopy (UBM) investigations and confirmed a permanent intrascleral space surrounding the T-Flux^®^ implant. On the other hand, the rationale for the absorbable implant is that it can maintain the surgically created intrascleral space for several months during the period of maximum postoperative inflammation and scarring. It is assumed that by the time the implant dissolves, the healing process is already completed [11,27,31]. Chiou *et al*. [31] investigated the scleral space by UBM several months after implantation of a collagen implant. They observed that the implant dissolved slowly, within 6 to 9 months, and was replaced by new autologous scleral tissue, yet leaving a tunnel. The lifespan of SKGEL^®^ is not clear but it is estimated to be at least as long as for the collagen implant. Marchini and associates [25] were unable to determine the lifespan of the SKGEL^®^ as it is nearly undetectable by UBM, in contrast to a collagen implant. In glaucoma revision surgery we extracted a hardly dissolved SKGEL^®^ device which we had placed there more than one year before.

In our patients, preoperative mean IOP was lower than in some of the studies cited. Ten patients in the SKGEL^®^ group and 12 patients in the T-Flux^®^ group had IOP meeting the criteria of a “qualified” success even before surgery. In these cases, indications for surgery were borderline IOP and progression of glaucoma despite an IOP below or equal to 21 mm Hg. Of those patients with a preoperative IOP <21 mm Hg, there was one patient in each group who still required medical glaucoma therapy after surgery. Setting a stricter cut-off IOP of <16 mmHg to define success, 10 patients (58.8%) in the T-Flux^®^ group and 9 (52.9%) patients in the SKGEL^®^ group showed a qualified success and 9 (52.9%) and 7 patients (41.1%) respectively, a complete success. We consider these results as being comparable for both groups.

Concerning complications during surgery, we could finish all interventions as planned; none of them had to be transformed to a trabeculectomy despite microperforations of the trabeculo-Descemet’s membrane. Postoperatively we did not see any iris incarceration. During the postoperative period there was no need for goniopuncture or needling, and no antifibrotic agents were employed, unlike in other studies [2,17]. In one patient a cyclocryocoagulation was performed for an uncontrollable rise in IOP. Most of our patients did not require postoperative antiglaucoma treatment; in less than 20% per group a permanent medical treatment was necessary after surgery.

Phacoemulsification can be done simultaneously in glaucoma patients suffering from additional cataract, avoiding the need of a second surgery. However, the contribution of lens removal to the IOP-lowering effect of a combined cataract-glaucoma surgery is unclear. Due to the fact that all eyes underwent phacoemulsification in our study, all eyes did benefit from a possible IOP-lowering effect. A cataract extraction without filtering surgery may result in a decrease of IOP in healthy eyes and to a lesser extent in some eyes with glaucoma [21,22]. Lens extraction may cause an increase in the depth of both the central and the peripheral anterior chamber. Accordingly, D’Eliseo *et al*. [23] observed that deep sclerectomy with SKGEL^®^ combined with phacoemulsification achieved better postoperative long-term results in IOP control than deep sclerectomy with SKGEL^®^ alone. Furthermore, phacoemulsification seems to lower the risk of iris adherence at the site of the Descemetic window, thus lowering the risk of an internal filtration block. However, other authors reported on the absence of statistically significant differences of IOP in patients having had non-penetrating procedures (deep sclerectomy, viscocanalostomy) with or without phacoemulsification, even in the long term [9,19,29,32,33]. Wiermann *et al*. [29] compared the efficacy of SKGEL^®^ versus T-Flux^®^ with or without phacoemulsification and concluded that phacoemulsification does not seem to interfere with IOP-lowering final results. The majority of authors [9,20,23] used a two-site approach when performing phacoemulsification, having a clear corneal tunnel situated mostly temporally. We carried out a one-site approach phacoemulsification also employed by Wishart *et al*. [19]. The site of the tunnel in combined surgery is probably without any influence on either the IOP-lowering effect or the occurrence of side-effects [29,32,34].

In conclusion, the results of our retrospective analysis confirm the results of other studies, in that in open-angle glaucoma a deep sclerectomy with implantation of a device in combination with phacoemulsification lowers IOP in a clinically relevant way over a long period. The effect seems to be independent from the absorbable or nonabsorbable property of the implant, while the risks of a combined surgery are few when performed by an experienced surgeon. A prospective study should confirm our results, ideally adding a third group without any implant in order to assess the de facto need of a space maintaining implant.

## Figures and Tables

**Table 1:** Pre- and Postoperative Intraocular Pressure (IOP)

Time	SKGEL^®^		T-Flux^®^	
	n	IOP ± SD (mm Hg)	n	IOP ± SD (mm Hg)
Preop.	17	20.6 ± 7.3	17	19.9 ± 7.2
D 1	16	12.8 ± 8.3	17	9.2 ± 6.2
M 1	17	13.9 ± 4.2	17	12.8 ± 4.3
M 3	17	12.4 ± 4.4	17	12.7 ± 3.1
M 6	17	12.4 ± 3.3	17	12.2 ± 2.9
M 12	14	14.6 ± 5.0	14	14.4 ± 3.3
M 24	10	14.4 ± 5.7	11	15.6 ± 2.8
M 36	8	14.8 ± 5.0	8	14.9 ± 3.8
M 48	4	12.3 ± 3.8	4	12.8 ± 2.9
